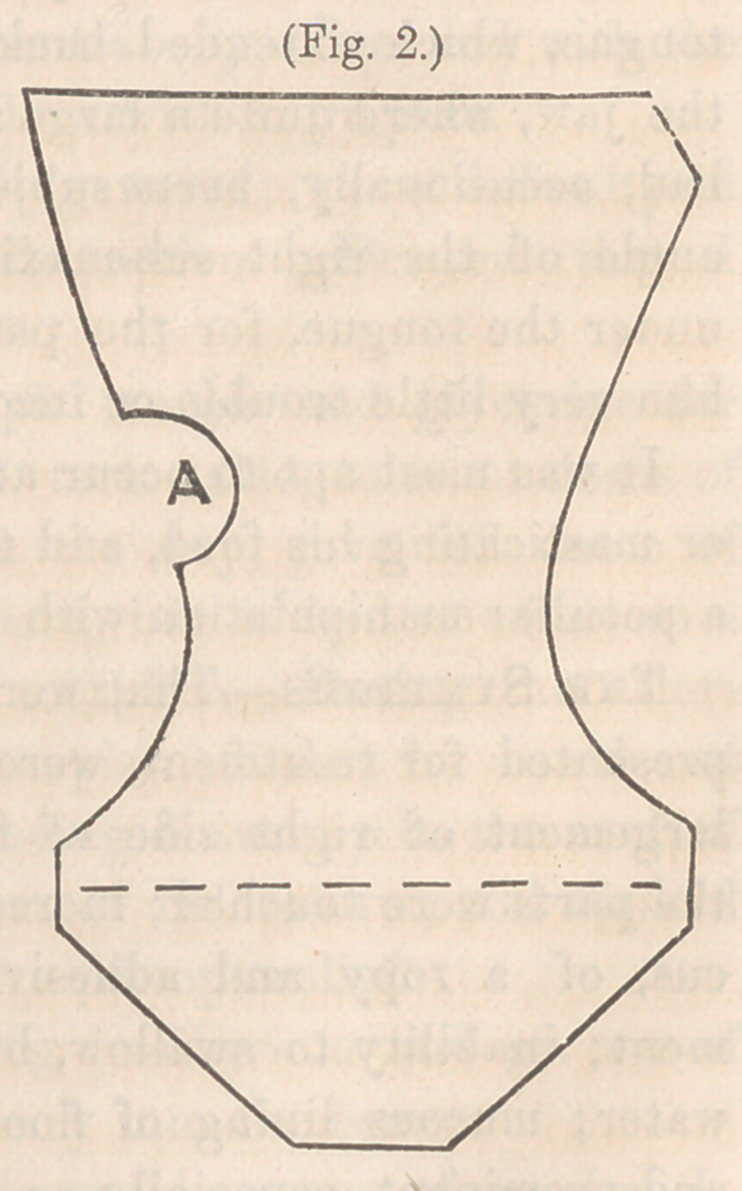# Report on the Use of Plaster-Paris in Fractures

**Published:** 1870-10

**Authors:** R. G. Bogue

**Affiliations:** Surgeon to Cook County Hospital, Chicago


ARTICLE XXXII.

### REPORT ON THE USE OF PLASTER-PARIS IN FRACTURES.

BY R. G. BOGUE, M.D., Surgeon to Cook County Hospital, Chicago.

PLASTER-PARIS IN FRACTURES.—The use of Plaster-Paris in surgery is not new. Its use in the treatment of fractures is not entirely of recent date. But its application in the manner recommended in this paper is not generally understood by the profession.

In presenting it to the notice of this Society, I do not bring it up as a new thing; nor as original with myself; but, having had my attention called to it, some three years since, and, having used it somewhat extensively during that time, and finding it to meet the requirements better than any thing else, is my excuse for calling the attention of the members of this Society to its use. Perhaps some use it already; if so, it needs no recommendation to them;—others may be induced to try it. I am sure that a more general knowledge of its use will establish it as a valuable addition to our means of treating fractures.

For a fuller and more detailed account of its use, we would refer to a paper, by Dr. James L. Little, of New York, in Vol. 18, of the Transactions American Medical Association; in fact, it was from this report that our attention was called to it. It came at a time when we had become weary in trying to treat fractures, of the leg especially, with the various appliances recommended; we could find none to fulfill the indications completely, nor even well, with comfort to the patient. But, with the Plaster-Paris, all the indications are met perfectly; so it seems to us. In our opinion, there is no style of dressing for fractures, in some localities, that can compare favorably with it.

It is cheap, light, readily applied, can be perfectly adapted to any-shaped limb, free from danger of producing injury to the skin by pressure, little or no danger of constricting or ligating the limb. From its perfect adaptation, it prevents much swelling, allows movement of the patient or part without detriment to the limb or dressing, secures perfect immobility of the bones at the point of fracture, admits of sufficiently free examination of the limb without disturbing the dressing, is worn with entire comfort, and keeps the limb dry by absorbing all exhalations from the skin.

The dressing may be applied directly after an injury; and, from its uniform support, prevents œdema. And, there is no danger to be feared from this, nor from pressure upon salient points, for the dressing can be applied so that there will be no more pressure upon the very point than on all the parts about it. There is no particular point which bears more pressure than another; the compression is even and equable.

In the treatment of fractures, there are several indications to be fulfilled: the essential one is the retention of the fragments in co-aptation; and the dressing which accomplishes that, most certainly is the best. Then, if, in addition, there is combined safety to the soft parts, and comfort to the patient, we have the essentials necessary for a good dressing.

In most cases of fracture, if there is maintained complete extension of the limb, there will be co-aptation of the fragments; and, if the parts can be thus secured, and all motion at the point of injury prevented, there will be little, if any, pain, and the parts be in the most favorable condition for early union. Take, for example, a fractured leg, and wherein is this dressing better than any other? By its taking hold of the foot and ankle below and the head of the tibia above, there is abundant means for making extension and counter-extension; all of the salient points are grasped so evenly that there is no danger from pressure. Then, the limb is nearly surrounded by the splint in its whole length, and evenly supported by it. The dressing is hard, which, absolutely, prevents the lower part of the limb from being drawn toward the knee, preventing any displacement in that direction; and, while the foot and knee are held in relative position, and neither end of the limb capable of being moved without the other,—for the whole dressing and limb must move together and alike,—there can be no motion of the fragments at the point of injury, thereby allowing any amount of moving of the limb and body that may be necessary, without detriment to the part.

It is the only dressing which will bear, with any certainty, the rough usage in cases of delirium, which is not uncommon after fractures in the dissipated. With this, there is but little danger of injuring the limb. We have had cases where the limb has been thrashed about very roughly for several days, and even the patient standing and walking upon it without injury.

It is the best of dressing where the patient is to be transported, as is necessary in military practice, and not unfrequent in civil.

This dressing may be applied at any time during the treatment of a fracture: slight abrasions and vesications will heal readily under it, the plaster absorbing all of the exudation from such surfaces. It can be removed and a new one applied whenever necessary; but often one splint will be sufficient for the whole treatment.

MATERIAL FOR THE DRESSING.—Ground Plaster-Paris, water, canton flannel, and bandages. Preparation for dressing: shave or oil the limb, if it is hairy; cut from the canton flannel such a piece as will fit the limb; if for the leg, long enough to extend from the knee to the ball of the foot, and wide enough to envelope the foot and leg, except about an inch, or an inch-and-a-half, in front, which must be left uncovered by the dressing. One thickness will be sufficient, if for a child or youth; but two will be required for an adult. Thoroughly saturate the cloth in a solution of the plaster and water—equal parts of plaster and water, by measure. The plaster must be thoroughly mixed with the water. The solution, when well prepared, as above, will be about as thick as good cream.

ITS APPLICATION.—Apply this saturated cloth smoothly to the limb, and confine it with a bandage; not too tight, but snug enough to adapt it well to the part. While it is being applied, the limb should be held in extension, and, when on, it must be laid upon something where it will rest easily, and then held in position until it "sets,"—which will take from ten to fifteen minutes; at any rate, it must be held until it is hard enough to keep its place; then it may be left to itself, and, in course of a few hours, it will be entirely dry. It is well to lay the limb with the extremity a little elevated. After it is dry, the bandage may be removed and another applied; but, if the limb is comfortable, there is no need of removing it until the part is to be examined, which should be done in the course of a few days. But, if there should be pain, it must be undone and so fixed as to relieve it. Our experience has been, that when the limb has been held well extended, and the fragments well co-aptated, there is entire freedom from pain as soon as the dressing is applied.

The best thing to lay the limb upon while drying is a feather pillow, being careful not to have the heel bear more weight than the rest of the limb.

Precautions.—The plaster dressing must not surround the whole limb, but at least an inch or two inches must be left uncovered to prevent constriction, and to allow examination; then, as the œdema disappears, the splint can be tightened up to the limb by re-bandaging. The sides of the splint will give sufficient for this. Never pad or wad inside of the splint to make it fit; but, if for any reason, it does not fit, remove it and apply another.

The fragments must be held in place, and the whole limb in proper position, while it hardens.

Usually, cold water should be used to mix the plaster; but, if it is desirable that the plaster set more quickly, then warm water may be used; and if a little common salt is added, it sets very rapidly.

Canton flannel is the best to use, for it requires fewer pieces to render the dressing firm enough than plain cloth.

This dressing is applicable to all fractures of the leg, arm, fore-arm, hand, fingers, and about the knee, ankle, and elbow joints. It may be applied to the thigh, but it is not as good for that as the dressing known as Buck's method, by extension. But, wherever there are salient points for the dressing to be applied to, to hold the parts at rest, it seems to us to be the best dressing that can be used.

We have never used it for fracture of the patella, but would think the fragments could be held in place better with it than with most any other, by encasing the front and sides of the knee and holding the fragments together with the hand until it hardens.

In case of compound fracture, there is to be only a portion of the side or sides of the splint cut out at the point of injury, and the bandage removed as soon as the splint is dry, and so fixed that the discharges may run over the edge of the splint instead of under it. This may be done by filling in at the edge of the opening, a little lint or cotton; then cover it and the skin about the wound with isinglass-plaster or collodium, or both.

This method of using Plaster-Paris must not be confounded with the "plaster-bandage," nor with the old method of encasing the whole limb with a plaster mould.

(Fig. 1.)

(Fig. 2.)

*Figure 1*—Is the form of the piece of cloth for the dressing. The heel should be laid on the middle of the dotted line *a b*, and the foot portion *c*, and leg portion *d*, folded to the bottom and sides of the foot and leg, lapping about the sides of the ankle, or foot, as necessary to have it fit smoothly; then it is ready to be confined with a roller.

*Figure 2*—Shows a piece cut out of the side in case of compound injury.

## Figures and Tables

**Fig. 1. f1:**
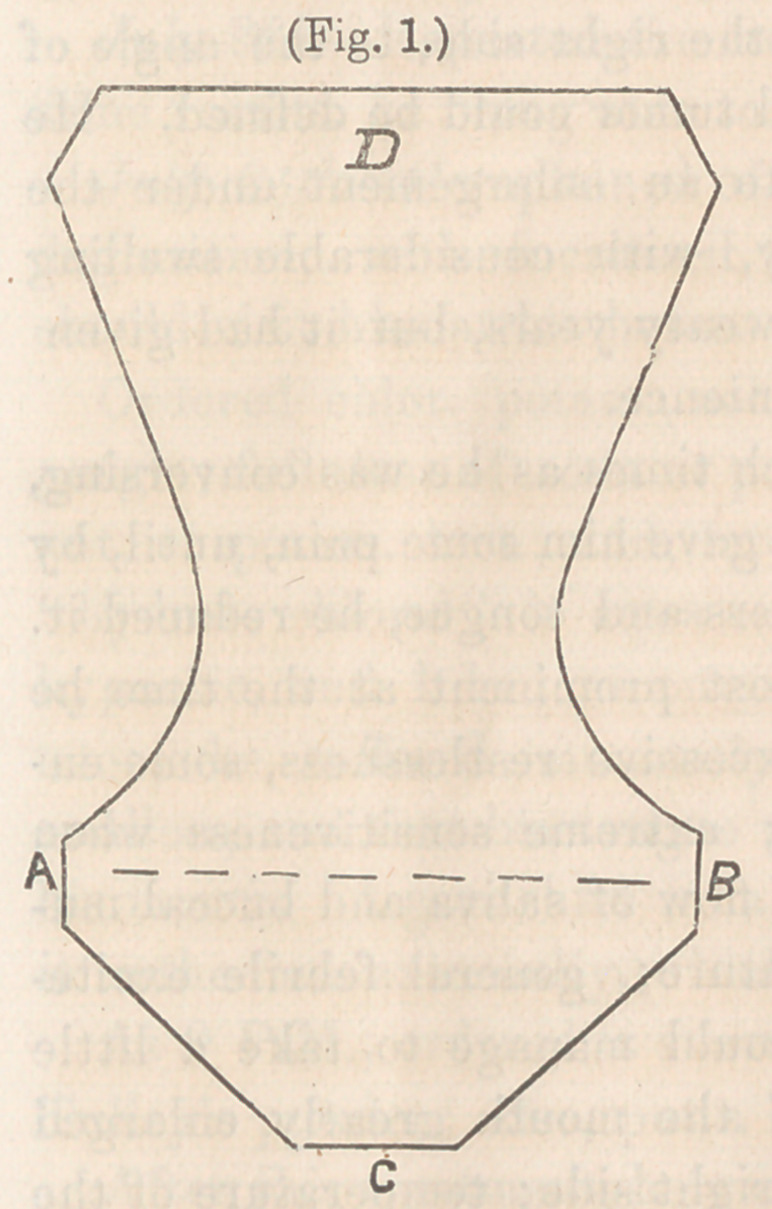


**Fig. 2. f2:**